# An Image Analysis Pipeline to Quantify Emerging Cracks in Materials or Adhesion Defects in Living Tissues

**DOI:** 10.21769/BioProtoc.3036

**Published:** 2018-10-05

**Authors:** Stéphane Verger, Guillaume Cerutti, Olivier Hamant

**Affiliations:** RDP, Université de Lyon, ENS de Lyon, UCB Lyon 1, CNRS, INRA, INRIA, Lyon, France

**Keywords:** Cracks, Cell adhesion, Image analysis, Mechanical properties, Tension, Github

## Abstract

Microcracks in materials reflect their mechanical properties. The quantification of the number or orientation of such cracks is thus essential in many fields, including engineering and geology. In biology, cracks in soft tissues can reflect adhesion defects, and the analysis of their pattern can help to deduce the magnitude and orientation of tensions in organs and tissues. Here, we describe a semi-automatic method amenable to analyze cell separations occurring in the epidermis of *Arabidopsis thaliana* seedlings. Our protocol is applicable to any image exhibiting small cracks, and thus also adapted to the analysis of emerging cracks in animal tissues and materials.

## Background

Microcracks are present in most materials; their number and extent generally increase when repeated stress is applied or when the temperature fluctuates, causing material fatigue and eventually, failure. Microcracks can reveal the magnitude and direction of the principal stresses that the material is experiencing. This property is widely used in mechanical engineering and geology (*e.g.*, Kowallis and Wang, 1983; Kranz, 1983; Joseph *et al.*, 2002). Microcracks are also present in biological structures, like bones. As in any material, they can lead to rupture (fatigue fracture). Such cracks also trigger signaling cascades involving osteoblasts and osteoclasts, resulting in bone remodeling (*e.g.*, Mori and Burr, 1993). Cracks can also be observed in soft tissues, notably as a result of cell to cell adhesion defects. This is particularly obvious in plant tissues, where cells do not migrate or intercalate (*e.g.*, Bouton *et al.*, 2002). Although many tools have been developed to study cracks in material sciences, they are often adapted to analyze long and thin cracks (*e.g.*, Griffiths *et al.*, 2017); they have not been customized for the analysis of emerging cracks in soft biological tissues. Here we describe a pipeline that detects and analyzes such cracks.

Our pipeline is based on a script that segments regions exhibiting a clear-cut pixel intensity contrast corresponding to cell separations or cracks. In our original publication, we stained *Arabidopsis thaliana* seedlings with propidium iodide (see Verger *et al.*, 2018 for the staining procedure), a fluorescent molecule that specifically binds to pectins in the cell wall. After washing the dye off, the cell contours are clearly marked. In a wild-type plant, this reveals a continuous epidermis where cells are fully attached to one another. However, when performing the same staining on a mutant with cell adhesion defects, holes between cells are revealed: a bright signal between cells marks emerging separations between these cells (Verger *et al.*, 2018). The contrast between the cells and the cracks is in fact strong enough to detect and quantify cell separations. After segmenting these cracks, a principal component analysis is performed on each of these segmented areas, yielding various information: area of the crack as well as its principal orientation (angle of the crack) and the shape anisotropy (derived from the eigen values and vectors calculated in the principal component analysis of the crack shape). These data can then be compared for multiple samples and sample series. In principle, other staining method may be used on any types of tissues or materials, as long as the contrast is strong enough for the script to detect the cracks.

## Software

1. Fiji program (http://fiji.sc/)
  Open-source plugin-based image analysis software based on ImageJ (https://imagej.nih.gov/ij/)
2. Python 2.7 (Interpreted high-level programming language for general purpose programming) (https://www.python.org/)
3. Python modules:
  - Matplotlib (2D visualization and data plotting library) (https://matplotlib.org/)
    Nose (Python unit test framework) (http://nose.readthedocs.io/)
  - Numpy (N-dimensional linear algebra library) (www.numpy.org/)
  - Pandas (Data manipulation and analysis library) (https://pandas.pydata.org/)
  - Pillow (Image manipulation library) (https://pillow.readthedocs.io/)
  - Pycircstat (Circular statistics library) (https://github.com/circstat/pycircstat/)
  - Scipy (Scientific computing library) (https://www.scipy.org/)
4. Open source package management and environment management system
  - Miniconda: https://conda.io/docs/
  - LINUX: https://repo.continuum.io/miniconda/Miniconda2-latest-Linux-x86_64.sh
  - MAC: https://repo.continuum.io/miniconda/Miniconda2-latest-MacOSX-x86_64.sh
  - Windows: https://repo.continuum.io/miniconda/Miniconda2-latest-Windows-x86_64.exe
5. Cell Separation Image Analysis Pipeline (Image analysis script described in this protocol) (https://github.com/sverger/Cell_separation_analysis)
  *Note: See “Installation procedure” in “Procedure” for the installation of Software 2 to 5*.

## Procedure

**Figure 1:**
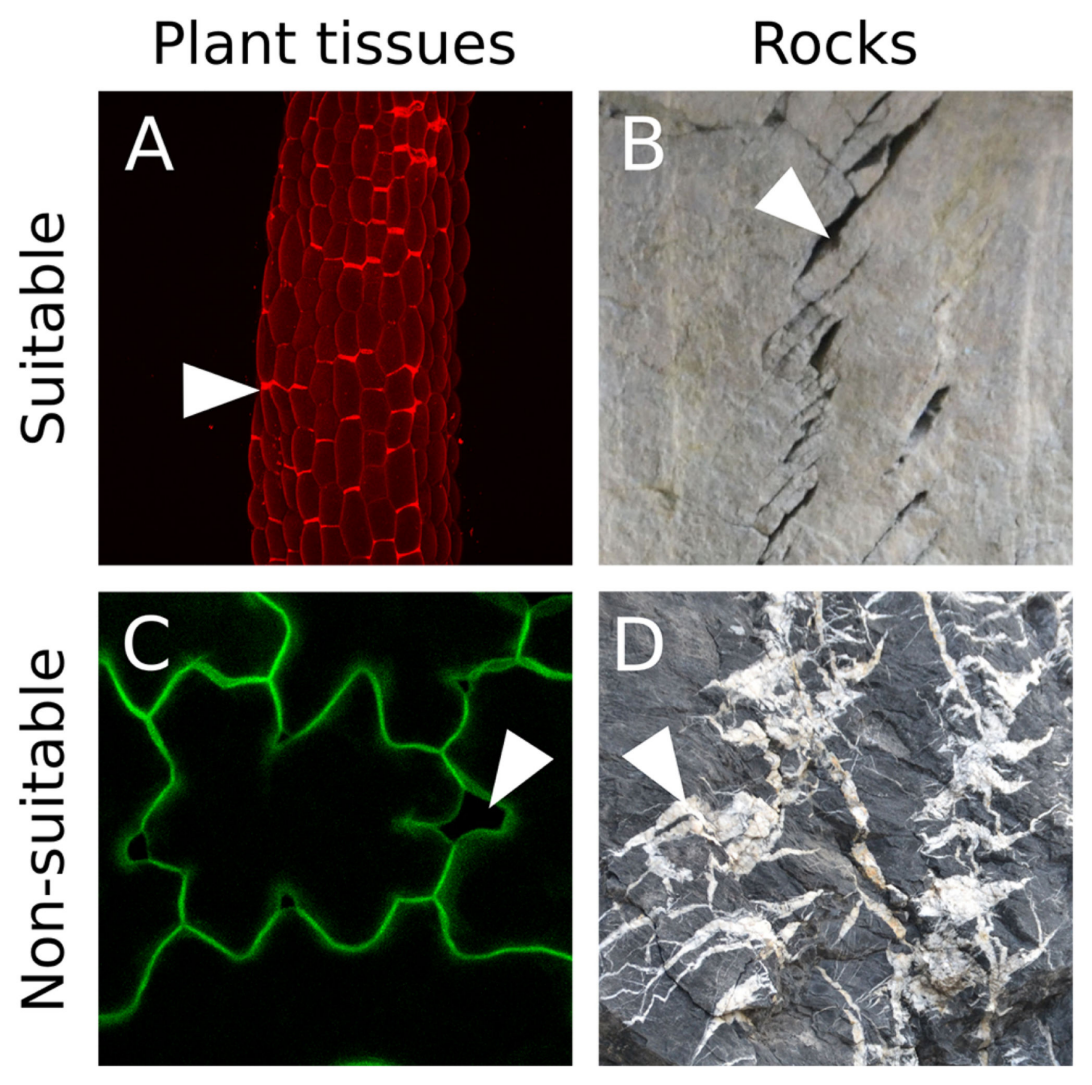
Suitable image type. A. Z-projection (maximal intensity) of a confocal image stack from a propidium iodide stained light-grown hypocotyl from the *qua1-1* mutant with cell adhesion defects (see Verger *et al.*, 2018). B. Cracks in rocks (credit photo: Pierre Thomas). In A and B the cracks are marked by a high pixel intensity contrast and form closed domains. C. Z-projection (maximal intensity) of a confocal image stack from a *qua1-1 pPDF1::mCit:KA1* (plasma membrane reporter) cotyledon epidermis (see Verger *et al.*, 2018). Although such fluorescent reporter can provide suitable images for our analysis, in this particular case the contrast between the cracks and the cell content is too low to allow a segmentation of the cracks with our pipeline. D. Picture of cracks in rocks (credit photo: Pierre Thomas). In this case the cracks do not form closed domains as most of them overlap. In addition, the pixel intensity is very variable throughout the picture such that some cracks do not exhibit a strong differential in pixel intensity. White arrowheads point to examples of cracks or cell separations in these images.

**Figure 2:**
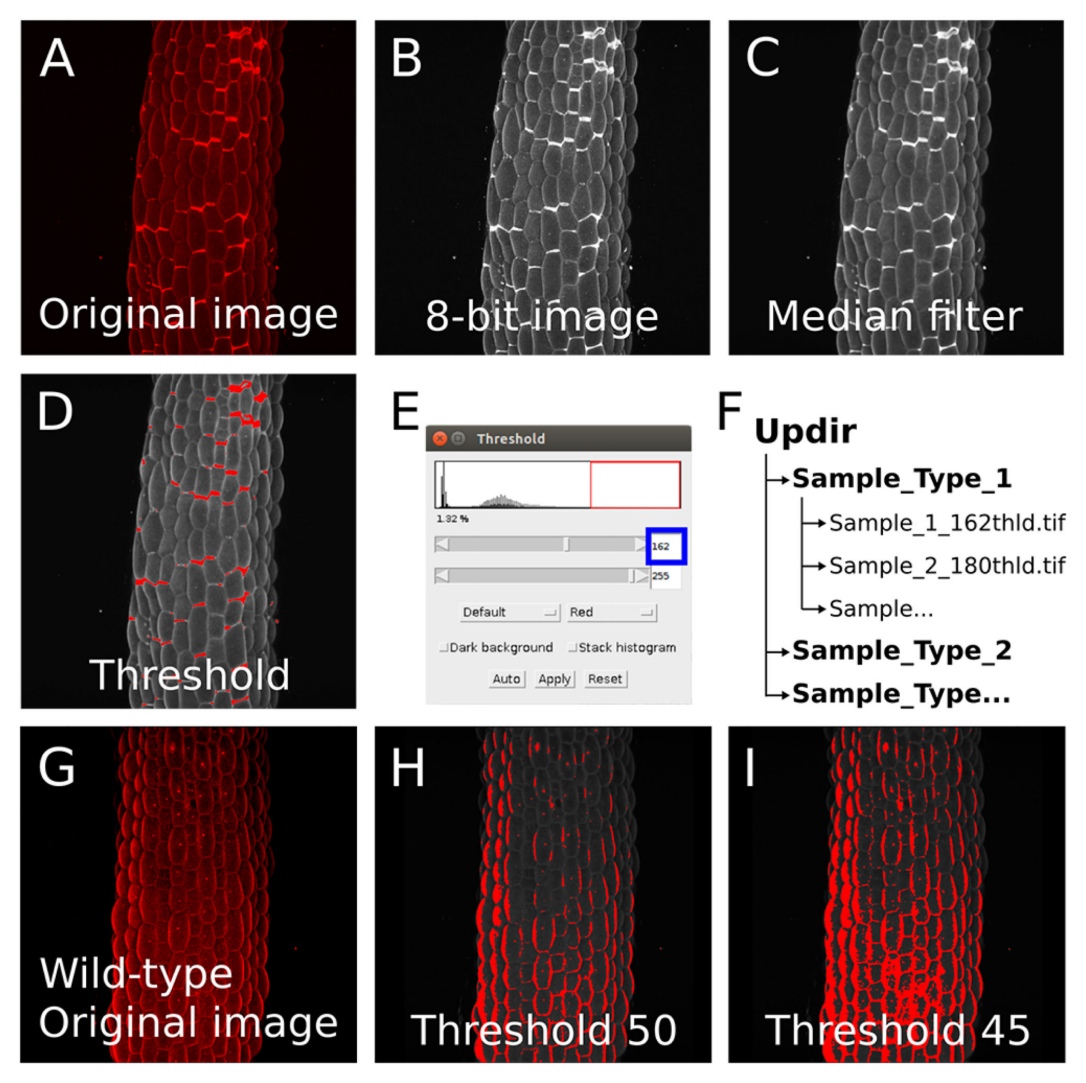
Image preprocessing in Fiji. A. Z-projection (maximal intensity) of a confocal image stack from a propidium iodide stained light-grown hypocotyl from a *qua1-1* mutant with cell adhesion defects (see Verger *et al.*, 2018). B-C. Images are preprocessed in imageJ: The image is converted to 8-bit (B) and a median blur with a radius of 3 is applied to reduce the noise and ease the segmentation (C). D-E. The threshold tool is used to determine the suitable threshold for segmentation in the pipeline. (D) Red zones will be segmented as cracks. (E) The threshold is adjusted in order to segment the cracks properly (*e.g.*, here to a value of 162). F. Example of file arborescence required for the pipeline to process the image series. G. Z-projection (maximal intensity) of a confocal image stack from a propidium iodide stained light-grown hypocotyl from a wild-type seedling showing no cell adhesion defects and thus not suitable to detect cracks with our pipeline (see Verger *et al.*, 2018). H-I. Because the differences in pixel intensity are locally small (unlike the *qua1-1* mutant with bright cell separation signals in panel A-D), we are unable to segment properly the image either with a threshold value of 50 (H) or 45 (I) as an example.

**Figure 3:**
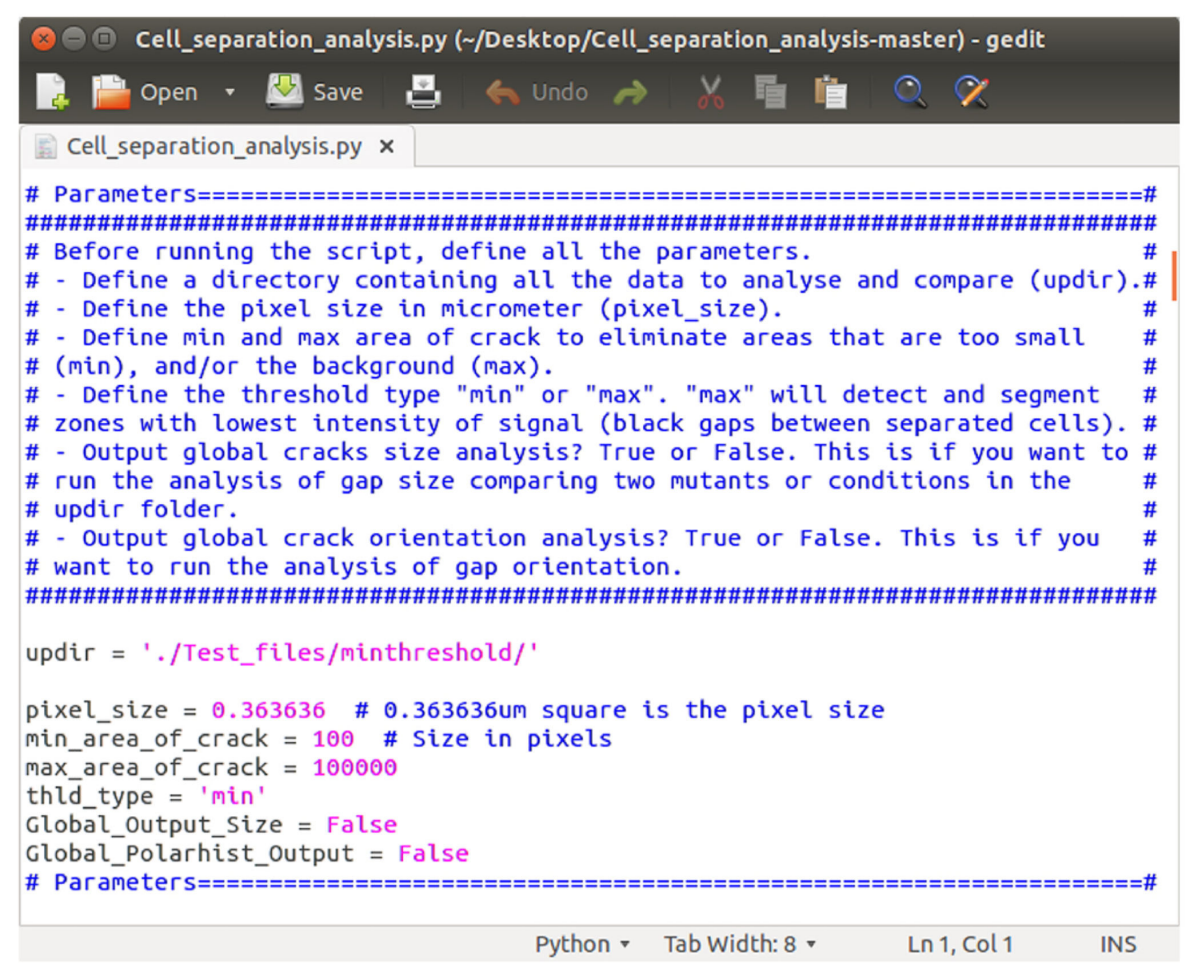
Parameter settings. Cell_separation_analysis.py Python script opens in a text editor, displaying the “parameters” section. The parameters can be modified according to your own requirements and the file can be saved before running the script.

- Image acquisition
  Our image analysis pipeline was developed to detect and quantify cell separations in plant epidermis. Such images can be obtained using a confocal microscope and by either staining the cell wall or cell contour with a fluorescent dye, or by imaging plants expressing a fluorescent reporter of the cell contours (typically, a protein at the plasma membrane). Z-Stacks can be obtained and projected in 2D (*e.g.*, using Fiji, max intensity). It is however crucial to obtain images in which there is a strong contrast between the cells and the “cracks” (*i.e.*, the zone where cells are separated. In Figure 1C the regions corresponding to cells are of comparable pixel intensity as the gaps between cells, making it impossible to automatically distinguish them). Furthermore, because our pipeline works by segmenting the whole cracks, the cracks need to form a closed domain (For example, in Figure 1D, the joints marking the cracks (white zones), overlap with one another, making it impossible to distinguish them individually with our pipeline). Thus our pipeline can in principle work with any 2D grayscale image containing clear-cut closed cracks (see examples of suitable and non-suitable images in Figure 1).
- Prerequisite: Image quality, preprocessing and threshold for “crack detection” in Fiji
  In order to determine if your images are suitable for this image analysis pipeline you need to make sure that the cracks will be properly segmented by pixel intensity:
  1. Load your 2D image in Fiji (Software 1).
  2. Change your image type to 8-bit. Image > Type > 8-bit (Figure 2B) and/or pick the right channel from a multichannel (*e.g.*, RGB) image (Image > Color > Split channels).
  3. Optionally you may enhance the contrasts of your image using the “Enhance Contrast…” function (Process > Enhance Contrast…> Set “Saturated pixels” to 0.3%).
    *Note: You may use a different approach (e.g., increase exposition time or laser intensity during image acquisition) to obtain enough contrast to segment the cracks*.
  4. Smooth your image with a median filter to remove noise (Process > Filters > Median… [Figure 2C]). Set the radius to a suitable value to reduce the noise, without blurring the image too much. Here again, you may also use a different approach to reduce the noise of your image, as long as in the end, you obtain a smoother detection of the cracks.
  5. Using the “Threshold…” tool, determine the suitable threshold that best separates the cracks from the surrounding regions (Figures 2D and 2E).
    *CRITICAL STEP: Depending on the nature and quality of your image, it may be difficult or impossible to segment the cracks based on a pixel intensity threshold. If most of your images are in this situation, this image analysis pipeline is not adapted to your study*.
  6. If you are able to properly separate the cracks from the surrounding regions, you may proceed with the analysis. Save your image in .tif or .jpg (File > Save As > Tiff… or JPEG…) and add ”_XXXthld” at the end of the name (where XXX is the threshold value previously determined in Step B5 as suitable to segment the cracks (*e.g.*, “sample_1_162thld.tif”. See Figures 2D-2F). The value before “thld” is the threshold value that will be used for the image segmentation later on (it has to be three digit long).
  7. You can pre-process as many images as you need before going further with the analysis. They will all be automatically processed if they are grouped in a folder. Note that in order to quantify and compare cracks orientation in multiple images, the images should be in a consistent orientation. The corresponding arborescence has to be organized as follows: A “main” directory (later on referred as “updir” in the script), containing subdirectories (*e.g.*, different mutants or growth conditions), each containing all the corresponding images (Figure 2F).
- Installation procedure
  You will need to have python (Software 2) installed on your computer in order to run the script (Software 5). The script has been designed, and should thus run properly, with python 2.7. You can then either directly run the script if you already have all the required dependencies (Software 3) in your python environment or install all the required dependencies in your python environment. If you do not have Python installed, or do not wish to interfere with your current Python environment, proceed with the steps below for our recommended installation using miniconda (Software 4), which will install python and all the required dependencies.
  1. Download the miniconda installer from the official website (link below “Software 4”, or here LINUX, MAC and WINDOWS). Alternatively you can use wget to perform this download from a terminal (LINUX or MAC). Open a new terminal window and run the command line below:
    For LINUX:
    *wget* *https://repo.continuum.io/miniconda/Miniconda2-latest-Linux-x86_64.sh* 
    For MAC:
    *wget* *https://repo.continuum.io/miniconda/Miniconda2-latest-MacOSX-x86_64.sh* 
  2. Install miniconda by running the installer:
    For LINUX and MAC: Open a new terminal window, navigate to the directory where you downloaded the installer (most likely in your “Downloads” folder. *e.g.*, *cd path/to/Downlaods/.*
    *Note: Input the actual path that leads to the folder “/Downlaods/”*) and run:
    For LINUX:
    *bash Miniconda2-latest-Linux-x86_64.sh* 
    *rm Miniconda2-latest-Linux-x86_64.sh* 
    For MAC:
    *bash Miniconda2-latest-MacOSX-x86_64.sh* 
    *rm Miniconda2-latest-MacOSX-x86_64.sh* 
    For WINDOWS: Execute the installer and follow the instructions.
    During the installation (LINUX, MAC and WINDOWS) you will be asked a number of choices. You can set the directory of your choice when asked (*e.g.*, ~/.miniconda). Make sure to answer YES when asked to add conda to your PATH.
  3. At this point, you should have miniconda installed. Test your installation by closing your current terminal window and running conda in a new terminal to make sure the command is found:
    *conda* 
  4. Download and extract the “cell_separation_analysis” repository from Github (following the link in Software 5, or here). On the Github page, click on the “Clone or download” green button at the right of the page. Then download and extract the zip.
    *Note: This folder contains the script (“Cell_separation_analysis.py”), a dependencies installation file for miniconda (“cell-sep-env.yml”) and test sample images (“Test_files”).* 
  5. In a terminal, navigate to the “/cell_separation_analysis-master” folder that you have extracted.
    *cd path/to/Cell_separation_analysis-master/* 
    *Note: Input the actual path that leads to the folder “/Cell_separation_analysis-master”.* 
  6. In the same terminal, create a new conda environment using the provided YAML file that lists all the software dependencies:
    *conda env create -f cell-sep-env.yml* 
    *Note: This will install Python and all the required dependencies. It may take a few minutes to complete the installation.* 
  7. When the installation is complete, in the same terminal, activate the environment:
    *source activate cell-sep-env* 
    *Note: You will have to run this command every time you want to use the cell separation analysis program, just after opening a new terminal window.* 
  8. You can then check your installation and whether the script runs properly by running the script on our test images. In a terminal, run:
    *ipython* 
    This will launch ipython. In ipython, navigate to the “/cell_separation_analysis-master” folder that you have downloaded:
    *cd path/to/Cell_separation_analysis-master/* 
    In the python console, type:
    *%run Cell_separation_analysis.py* 
    This should run the script and generate its output in the “/Test_files” folder of “/Cell_separation_analysis-master”.
- Running the script
  In order to run the script with your own images, you need to edit the “parameter” section of the script. To do so, open the “Cell_separation_analysis.py” file in a text editor. Scroll down to the section called “parameters” where there are 7 entries that you may modify (see Figure 3):
  1. Set the directory where you placed the images to analyze. Remember that your folder has to be organized in a specific way: A “main” directory corresponding here to “updir”, containing subdirectories (*e.g.*, different mutants or growth conditions), each containing all the corresponding images (Figure 2F). You can either enter the full path to the directory (*e.g.*, /Home/Path/to/updir/), or simply enter ./updir/ (where you replace “updir” by the actual name of your folder). In the latter case, you will have to navigate to the parent folder of your “updir” in Ipython before running the script (as described in Step C8).
  2. Set the pixel size. This is required in order to perform analyses of crack area. Usually, for confocal images this information can readily be found by loading the original image in Fiji and looking at its properties (Image > Properties… > Pixel width or height). The unit of length should be micron. For other types of images, you will need to determine the actual pixel size, for example in Fiji, using an internal scale (Analyze > Set Scale…).
  3. Determine the minimum and maximum area of crack. This step corresponds to a filter as it eliminates areas which are too small (“min_area_of_crack”, *e.g.*, background noise) as well as the global background of the image (“max_area_of_crack”, *e.g.*, the empty space around the tissue). These values are in pixel number. You may have to try several times with different values in order to determine the right parameters empirically.
  4. Set the threshold type. The cracks can be of either a higher or lower pixel intensity than the surrounding region. Thus the threshold can be set as “min” or max”. The “max” will detect and segment zones with lower signal intensity (*i.e.*, the crack is darker than the surrounding region) and the “min” will detect and segment zones with higher signal intensity (*i.e.*, the crack is lighter than the surrounding region).
  5. You may then decide to run more global analyses if you have multiple sample types (“Global_Output_Size”) and multiple images by sample type (Global_Polarhist_output). See “Data analysis” section to determine if you should run these analyses. These are by default set to “False” which mean they will not be performed. Replace “False” by “True” if you want them to be performed.
  6. Once all the parameters are correctly set, save the script and run it as described in Step C8.

## Data analysis

**Figure 4:**
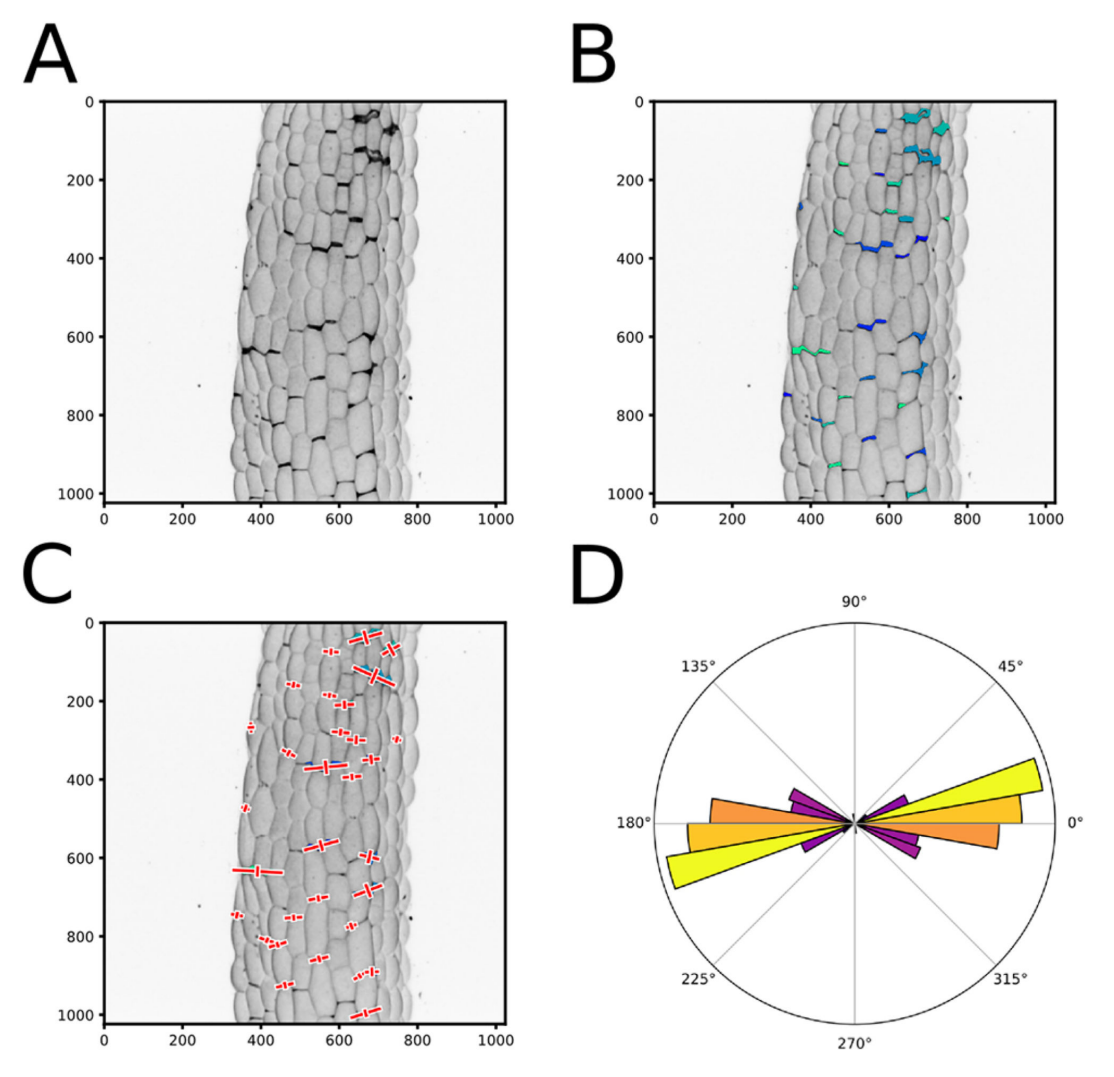
Output of the cell separation analysis pipeline. A. Pixel intensity inverted version of the image (Figure 2C). B. Same image as in (A) with an overlay of the segmented areas that are identified and labeled using different colors to ease visualization. C. Same image as in (B) with a representation of the vectors resulting from the principal component analysis of the crack shapes (Red crosses). To improve the visual output, the eigen vector that are mapped on the images are multiplied by a factor 2 and by the square root of the corresponding eigen value. D. A polar histogram representing the distribution of the crack orientations in an image. The square root of the principal eigen value for each plotted angle is added to normalize the angle value by its relative weight. A color map is used in the polar histograms representing the relative number of angles binned in each histogram bar (independently of their weight) where yellow is high and purple is low.

1. As explained in the background section, this script can generate different outputs. From the detected cracks in each image, the script will generate three images: a pixel intensity inverted version of the image, the same image with an overlay of the segmented areas, and the same image with an overlay of the anisotropy and principal angle of the area, directly saved as vectorial PDFs. It will also generate a .csv file containing, for each segmented cracks, the label number, center position, area in pixels and micrometer square, the main orientation (angle) of the crack, the shape anisotropy, the eigen values and vectors. Finally for each image, a polar histogram representing the distribution of crack orientations in the image is created and saved as a vectorial PDF (see output generated in the test of the script in the previous step and Figure 4).
2. If you process multiple images from one sample series (*e.g.*, technical or biological replicates), you can output a summary of their properties (see Step D5, “Global_Polarhist_Output = True”). It will generate a polar histogram representing the distribution of cracks orientation for all the images of a sample series pooled together, and save it as a vectorial PDF. It will also output a .txt file containing for each image the circular mean angle (between 0° and 180°), the resultant vector length (an estimation of the coordinated directionality of the cracks, between 0 and 1; a value of 0 means that the crack orientations are homogeneously distributed whereas, a value of 1 means that all the cracks have the same orientation) and the mean anisotropy of the crack shapes. At the end of the text file, a global analysis of all the images of a sample series is generated: circular mean angle, resultant vector length and shape anisotropy for the pooled values of all the images of the sample series. It will also output the result of an RAO’s spacing test, which assesses whether the angles are uniformly distributed (statistically no preferential angle orientations), or if there is a significant angular bias.
3. The script also offers the possibility to compare different type of samples. You can compare the total area of the cracks in two sets of samples (*e.g.*, 10 images of mutants 1 compared with 10 images from mutants 2) (see Step D5, “Global_Output_Size = True”). This will run statistical tests on the compared samples to determine if the average area of cracks in images of one sample series is different from that of another sample series. The choice of statistical test depends on the normality and the variance of the data. First, a Shapiro’s test for population normality is run on both sample series. If at least one of the sample series does not have a normally distributed population, a non-parametric Wilcoxon rank sum test is run to test whether the samples are statistically different. Otherwise, if both sample series are normally distributed, a Bartlett’s test for equal variances is run. If both sample series have equal variance, a Student’s *t*-test is run and otherwise a Welch’s *t*-test is run to test whether the samples are statistically different. A summary of these tests is saved as a .txt file, and a boxplot of this comparison is saved as a vectorial PDF.
4. You may then perform further analyses depending on your needs, using the different output files containing all the raw data.

## Notes

1. The most critical step in this protocol is to check the suitability of your images as described in “Procedure B”. If most of your images do not pass this test, this image analysis pipeline may not be suited for your study. Conversely, it is important to realize that it is also rare to be able to segment 100% of the objects that you would identify as cracks visually. You should then decide what is an acceptable yield in your case.
2. Following our recommended installation procedure you should in principle be able to run our image analysis pipeline on any system (LINUX, MAC, and WINDOWS). However, the script has so far only been tested on a computer running Ubuntu 14.04 and Python 2.7. We recommend testing the macro with our sample images (Step C8) and check if the output images look similar to what is reported in our original publication (Verger *et al.*, 2018).
